# Canadian Regulatory Perspective on Next Generation Risk Assessments for Pest Control Products and Industrial Chemicals

**DOI:** 10.3389/ftox.2021.748406

**Published:** 2021-11-04

**Authors:** Yadvinder Bhuller, Deborah Ramsingh, Marc Beal, Sunil Kulkarni, Matthew Gagne, Tara S Barton-Maclaren

**Affiliations:** ^1^ Health Evaluation Directorate, Pest Management Regulatory Agency, Health Canada, Ottawa, ON, Canada; ^2^ Safe Environments Directorate, Healthy Environments and Consumer Safety Branch, Health Canada, Ottawa, ON, Canada

**Keywords:** next generation risk assessment, integrated approach to testing and assessment, adverse outcome pathways, new approach methodologies, weight of evidence

## Abstract

In 2012, the Council of Canadian Academies published the expert panel on integrated testing of pesticide’s report titled: Integrating emerging technologies into chemical safety assessment. This report was prepared for the Government of Canada in response to a request from the Minister of Health and on behalf of the Pest Management Regulatory Agency. It examined the scientific status of the use of integrated testing strategies for the regulatory health risk assessment of pesticides while noting the data-rich/poor dichotomy that exists when comparing pesticide formulations to most industrial chemicals. It also noted that the adoption of integrated approaches to testing and assessment (IATA) strategies may refine and streamline testing of chemicals, as well as improve results in the future. Moreover, the experts expected to see an increase in the use of integrated testing strategies over the next decade, resulting in improved evidence-based decision-making. Subsequent to this report, there has been great advancements in IATA strategies, which includes the incorporation of adverse outcome pathways (AOPs) and new approach methodologies (NAMs). This perspective provides the first Canadian regulatory update on how Health Canada is also advancing the incorporation of alternative, non-animal strategies, using a weight of evidence approach, for the evaluation of pest control products and industrial chemicals. It will include specific initiatives and describe how this work is leading to the creation of next generation risk assessments. It also reflects Health Canada’s commitment towards implementing the 3Rs of animal testing: reduce, refine and replace the need for animal studies, whenever possible.

## Introduction

Evidence-based decision-making, rooted in robust scientific risk assessments, is paramount for the initial market-approval and subsequent evaluations of registered pest control products and industrial chemicals in Canada. The federal regulatory frameworks governing the life-cycle management of these products provides sufficient flexibility for the responsible regulatory authority to evaluate scientific studies from a wide variety of published and unpublished sources. It also provides an agile approach to considering alternative strategies to health risk assessments and incorporating non-animal technologies, when applicable, for hazard identification. The health risk assessment process itself, a function of both hazard and exposure, is well described in several documents and is aligned with international approaches. These include technical documents, describing program-specific decision-making frameworks (Health Canada, 2021a), and non-technical ones, such as Health Canada’s primer on scientific risk assessment (Saner, 2010). Further, in the area of industrial chemicals assessment, efforts have been made to advance the development and implementation of novel scientific assessment approaches through the publication of science approach documents (Health Canada, 2021b). Health Canada has also progressively introduced new methods to effectively identify and address substances of varying concern and continues to update their data requirements (Health Canada, 2013a) thereby enabling them to be well positioned to transition to next generation risk assessments (Krewski et al., 2014).

In 2012, the Council of Canadian Academies (CCA) published the expert panel report on integrating emerging technologies into chemical safety assessment (CCA, 2012). This report was prepared for the Government of Canada in response to a 2009 request from the Minister of Health and on behalf of the Pest Management Regulatory Agency (PMRA). It was the first Canadian report that provided the scientific status on integrated strategies and identified the potential paradigm shift for a more inclusive approach where integrated approaches to testing and assessment (IATA) go beyond using them just for data-poor chemicals (e.g., pesticide formulants and industrial chemicals). The report also included a 10-year vision for the evolution of IATA within the regulatory context and a foundational starting point that included these elements: using a common vocabulary, data platforms and standards, digitization of legacy data, international coordination, stakeholder communication, and functional collaboration. The CCA and other international reports, such as the National Research Council’s report (NRC 2007), have been pivotal in establishing the Canadian regulatory approach for identifying, exploring, and implementing IATAs. Some IATAs utilize adverse outcome pathways (AOPs) and more recently new approach methodologies (NAMs). Publications, such as the 2020 article on toxicity testing in the 21st century (Krewski et al., 2020), provide insights on the advances in biological sciences and how these have led to this ongoing paradigm shift. Future perspectives on the continued evolution of toxicity testing to strengthen regulatory risk assessment are also noted, which includes ensuring that any alternative approach adheres to the established health and safety standards required for these products.

This article now provides the first Canadian regulatory update on how the regulatory authorities responsible for pest control products and industrial chemicals are advancing the incorporation of alternative and non-animal strategies. It demonstrates how these program areas have successfully positioned themselves for the next generation of risk assessments by elaborating on early conceptual frameworks. References to recent and key publications are provided along with insights on how these areas have been contributing to this paradigm shift through the establishment and successful maintenance of a strong, multi-stakeholder collaborative approach.

## Regulation of Pest Control Products and Industrial Chemicals

Chemical substances, which includes pest control products and industrial chemicals, are stringently regulated in Canada to protect human health and the environment (Health Canada, 2017a). While Health Canada is the responsible federal department for the market approval and subsequent oversight of pest control products and industrial chemicals, there are two program areas that are accountable for this work. Specifically, Health Canada’s PMRA is responsible for pesticide regulation in Canada while, in part, the Healthy Environments and Consumer Safety Branch (HECSB) in collaboration with Environment and Climate Change Canada is responsible for industrial chemicals.

Under authority of the Pest Control Products Act, Health Canada registers pesticides after a stringent, science-based risk assessment, re-evaluates pesticides on the market on a cyclical basis, and is actively involved in national and international science-policy initiatives. As noted in the 2019–2020 annual report, PMRA continues to evaluate pesticides in cooperation with other jurisdictions and over the last 2 years, the Agency’s focus has been on a major transformation of its pesticides program (Health Canada, 2021c). The latter is exploring a further integration of the pre- and post-market activities, which includes incorporation of next generation approaches to risk assessment.

The Canadian Environmental Protection Act, 1999 (CEPA, 2019; CEPA) provides the legislative framework for industrial chemicals, including new chemical substances (domestic and imports) as well as substances that are currently on the Canadian market (i.e., existing substances). Leading the world in chemicals management, Canada was the first to systematically categorize or prioritize the 23,000 substances on the Domestic Substances List (DSL) for risk assessment, initiating the Chemicals Management Plan (CMP) in 2006 (Health Canada, 2016a). Risk assessments of the approximate 4,300 priority chemicals were conducted over three phases (2006–2021) and required the development of new methodologies and scientific approaches to continue to effectively deliver an evolving risk assessment program. For industrial chemicals, there is a range of toxicity data available, from data-rich to data-poor, and an ongoing need to prioritize, assess and manage diverse and increasingly complex substances and mixtures. The Government of Canada is also building on the successes of the CMP to renew its approach to chemicals management including follow-up considerations on the report from the House of Commons Standing Committee on Environment and Sustainable Development on the statutory review of CEPA (Environment and Climate Change Canada, 2018).

## Modernizing Approaches to Risk Assessment

In comparison to industrial chemicals, pesticides and pest control products are considered data-rich chemicals. The regulatory submissions rely on a prescribed list of data requirements that include several animal studies and often comprise *in silico* (quantitative-structure activity relationship (QSAR)), *in vitro* assays, and more recently NAMs (e.g., defined approaches for skin sensitization (OECD, 2021a). Similarly, when considered equally or better suited to measure toxicity, alternate approaches, such as *in vitro* data, read-across using surrogate data, weight-of-evidence (WoE) for substance classes, and QSAR data from internationally accepted models, are examples of frequently accepted NAMs for industrial chemicals.

In contrast, there are no prescribed data requirements for existing substances, under CEPA, and assessments make use of best available data. Accordingly, the program has progressively advanced the use of NAMs from computational modelling, read-across and category approaches to more complex evidence integration approaches to identify and address emerging priority substances. Typically, a WoE approach is relied upon by evaluating the results from the alternative approaches along with the totality of evidence, which includes published information. Enriching evidence integration for WoE assessment has been supported through the development of IATA methodologies; endocrine activity has been one area of focus in this respect for the existing substances program at Health Canada. Workflows to assimilate data collected from traditional and NAM sources to generate predictions regarding potential endocrine disruption activity for a subset of chemicals of regulatory interest has illustrated that NAMs can be a protective approach for human health risk assessment (Webster et al., 2019).

The year 2022 marks a decade since the release of the CCA report and significant progress has been made on a variety of NAMs, which includes *in silico* based approaches. The latter has found the most widespread use and acceptance in regulatory data submission and assessment. To address existing substances in Canada, efforts have focused on validation exercises to increase confidence in the application of a suite of models for the DSL chemical space (Kulkarni and Barton-Maclaren 2014; Kulkarni et al., 2016) as well as contributing to imperative steps forward to promote international harmonization. Key developments have included progress on standardized *in silico* toxicology (IST) protocols (Myatt et al., 2018; Hasselgren et al., 2019), endorsement of OECD guidance for defined approaches to testing and assessment (OECD 2016a), and grouping of chemicals and read across (OECD 2017). Evolving these approaches further, cheminformatics-based methods for read-across of point of departures (PODs) are being explored to build confidence in quantitative read-across to specific endpoints (Yang et al., 2021).

Notably, *in vitro* and omics-based approaches are also being explored quite broadly across Health Canada. Specifically, transcriptomics data is currently used in a WoE to better understand chemical mode of action, justify read-across groupings, and fill data gaps (Yauk et al., 2019). Health Canada’s CMP phthalate assessment demonstrated that gene expression patterns could be used to support category development and the selection of specific compounds for cumulative risk assessment (Health Canada 2015). Transcriptomics also holds promise in the selection of PODs for prioritization and quantitative risk assessments. Results from recent case studies focused on flame retardants demonstrated that *in vitro* transcriptomics data, coupled with *in vitro* to *in vivo* extrapolation (IVIVE), provide PODs that are protective of human health and allow for potency ranking (Gannon et al., 2019; Rowan-Carroll et al., 2021). Similarly, quantitative high-throughput screening assays, that provide mechanistic and quantitative data across a broad toxicological space, also have established utility in the assessment of potential for human health risk. Specifically, a multi-agency retrospective case study conducted under the Accelerating the Pace of Chemical Risk Assessment (APCRA) initiative demonstrated that *in vitro* data from the ToxCast program, comprising nearly 1400 toxicological endpoints, could be used to derive points of departure for risk assessment activities (Paul Friedman et al., 2020). Building on the approach and learnings from the collaborative case study, Health Canada published a science approach document providing a rationale and guidance for how to apply the approach as an early screen of potential for risk in the context of the CMP (Health Canada, 2021d).

## Importance of Multi-Stakeholder Collaboration

As an OECD member, Health Canada is involved in several initiatives related to IATA, NAMs, and ongoing developments of several technical guidelines. An underlying reason for this international collaboration continues to be rooted in the 3R principles: reduce, refine and replace animal studies, when possible. However, another aspect is the mutual acceptance of data whereby harmonizing requirements provides a common basis for all authorities (OECD, 2021b).

To allow for broader acceptance of IATAs, NAMs, and no longer routinely requiring specific animal assays for toxicity testing, Health Canada continues to rely on the North American Free Trade Agreement (NAFTA) Technical Working Group on Pesticides (TWG) and the Canada-United States Regulatory Cooperation Council (Health Canada, 2016b; 2020; RCC). This cooperation has resulted in successful collaboration with stakeholders and global experts from all areas including Industry, Academia, and Non-Governmental Organizations. Health Canada’s participation also provides an opportunity to provide guidance so that outputs are fit-for-regulatory purpose and build regulatory, public, societal, and scientific confidence in NAMs. This is consistent with the 2018 Interagency Coordinating Committee on the Validation of Alternative Methods (ICCVAM) strategic roadmap for establishing new approaches to evaluate the safety of chemicals and medical products in the United States (ICCVAM, 2018). Individual project plans are also built upon the strategy noted in the CCA report by first focusing on retro-analysis and less complicated assays such as the acute toxicity studies (Health Canada, 2017b; Linke et al., 2017; Allen et al., 2021). The NAFTA TWG has also been used to develop science-policies, which are then brought for broader acceptance through OECD. For example, built on the NAFTA QSAR document (NAFTA TWG, 2012), which was primarily focused on pesticides, the OECD guidance document expanded to cover industrial chemicals with added focus on mechanistic considerations (OECD, 2015). Similarly, the NAFTA developmental neurotoxicity study guidance (NAFTA TWG, 2016) as well as PMRA’s guidance document for waiving or bridging of mammalian acute toxicity tests (Health Canada, 2013b) were also used as the foundational pieces for completed (OECD, 2016b) and/or ongoing OECD technical guidelines.

With parallel goals in mind, industrial chemicals have the additional pressures of lack of data, aggressive priority setting and assessment mandates. In turn, RCC has also played a role in advancing assessment methods for Health Canada’s industrial chemicals programs (Health Canada, 2017c), as has the OECD Hazard Assessment Programme related to the improvement and acceptance of approaches intended to minimize the need for animal testing. Foundational work upon which HECSB continues to build include concepts, guidance and lessons learned related to IATA (OECD, 2020; OECD, 2021c) and guidance on physiologically based kinetic models for regulatory purposes (OECD, 2021d). Considerable momentum for regulatory application of NAMs has been gained through research-regulatory partnerships, nationally and internationally, including regulatory, academic, and stakeholder communities. The APCRA network, co-led by the US EPA, Health Canada and the European Chemicals Agency (ECHA), is another example of a successful collaboration between international and intergovernmental bodies (Kavlock et al., 2018). The Friedman et al. and Health Canada work highlighted above are examples of complete progression from collaboration to development of a Canadian-specific approach. It is important to also note that partnerships between risk assessment and research experts to achieve the goal of demonstrating robustness, reliability and readiness of non-animal based approaches in regulatory applications is also a model of interest beyond the chemicals assessment community (Chauhan et al., 2021).

## Mobilizing Teams and Establishing the Regulatory Pivot

The transition from exclusively relying upon conventional testing approaches to inclusion of NAMs requires a high level of engagement and collaboration given the pivot required to consider incorporating such approaches in regulatory decision-making. Specifically, some complex issues to address include validation, interpretation and application frameworks, guidelines for NAMs or other disruptive technologies, and ethical considerations for using big data (Mittelstadt and Floridi, 2016). There are also legal considerations along with how the public and society will view this transition. While these areas are beyond the scope of this perspective, they continue to be part of ongoing discussions. This section will now focus on the approaches used to mobilize Health Canada scientists.

**FIGURE 1:**
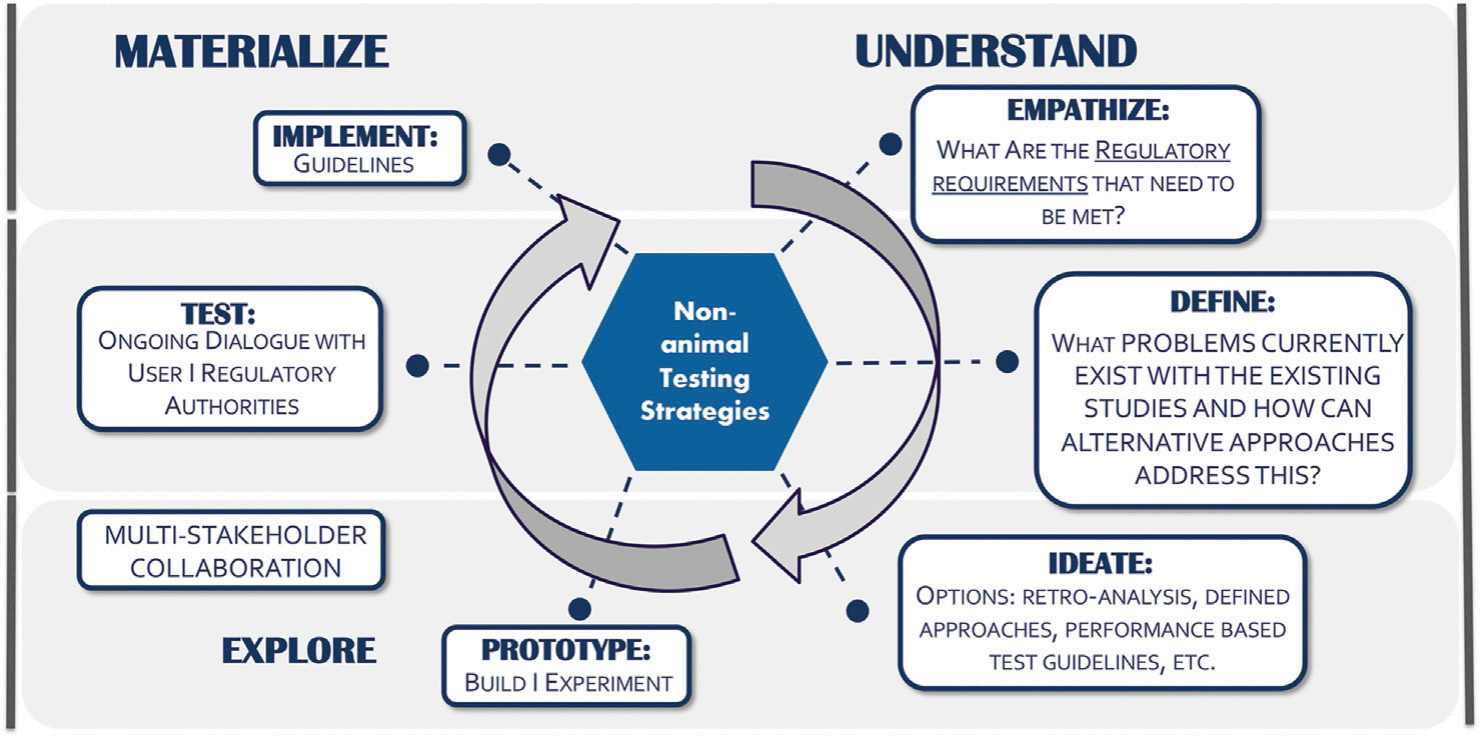
Non-animal testing approaches: Using design thinking.

The model used to engage regulatory scientists and establish the pivot for exploring non-animal testing strategies has relied upon an adaptation of the design-thinking approach (Figure 1). Briefly, a top-down approach that relies on the user experience (UX) with conventional assays required for regulatory purposes is the starting point. This insight is then incorporated from concept through to application using a process that understands the data gaps/uncertainties, explores approaches through collaboration, and materializes by learning from successes and failures from the UX perspective. The implementation is then achieved through publication to allow for broader distribution and potentially acceptance of the alternative approach.

**FIGURE 2:**
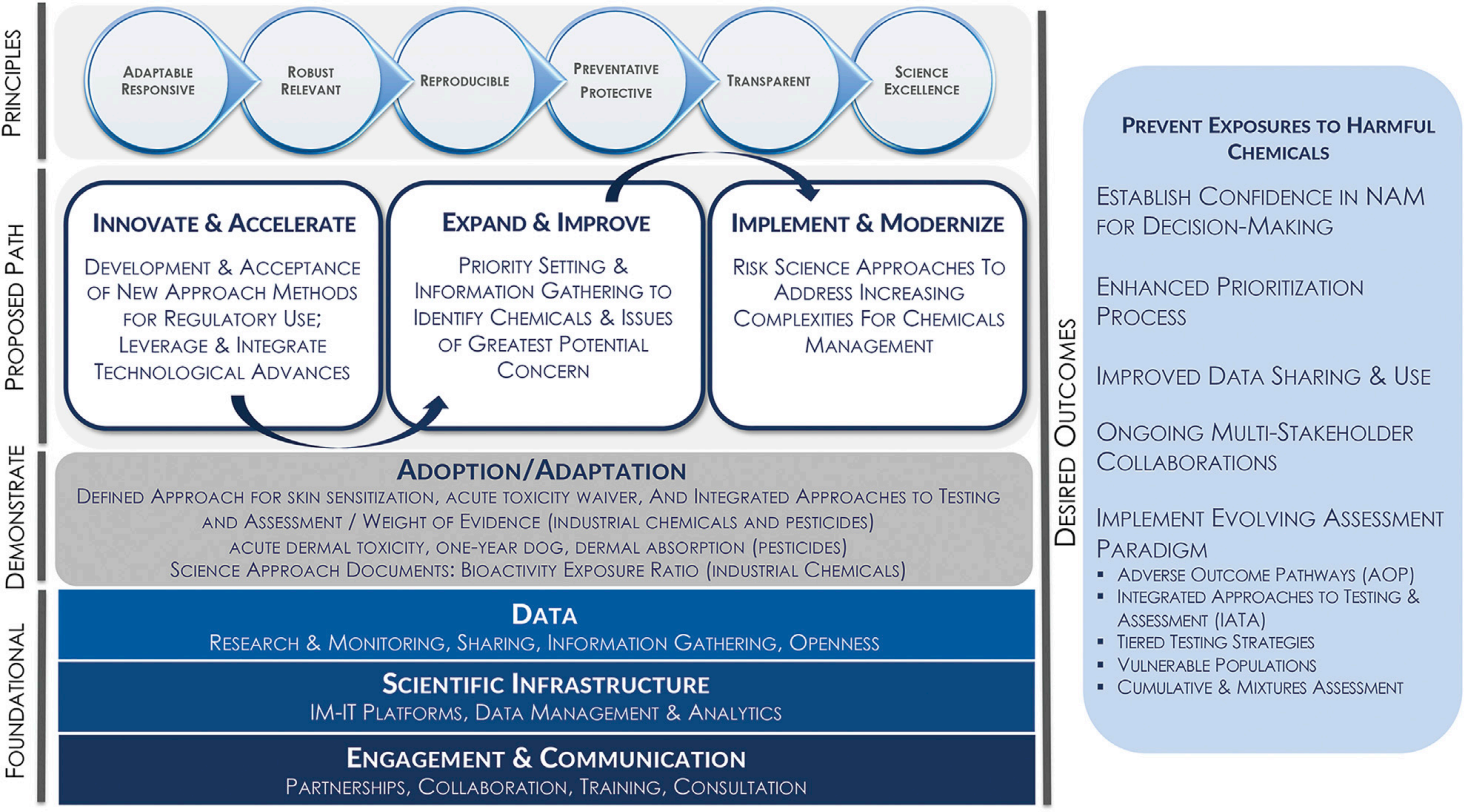
Innovate and accelerate use of NAMs: Translating case study findings into applications.

Translating case study findings into applications, using a framework that incorporates both innovation and acceleration, has also been extremely useful in the exploration and implementation of NAMs (Figure 2). Through the use of practical case studies designed to address specific regulatory needs, methods can be informed by proof of concept research and lessons learned to develop best practices and guidance for the application of fit-for-purpose approaches. Consistent with focused efforts internationally, Health Canada has as an objective to enhance innovation and risk assessment modernization to maintain a world-class chemicals management program. The overarching program and risk assessment principles that have been key for success to date must be reinforced and incorporated to effectively provision the proposed path toward modernization. A multi-pillar approach is envisioned for the transition to modernization of some elements of the program through the accelerated development and acceptance of new methods, taking into consideration a wide range of use and decision contexts. Importantly, the aim is to bring all of these elements together in order to use the most relevant data for the protection of human health and the environment.

## Discussion and Next Steps

This perspective provides the first Canadian regulatory update on how Health Canada is advancing the incorporation of alternative, non-animal strategies for the evaluation of pest control products and industrial chemicals. It includes specific, multi-stakeholder initiatives that are aligned with the Department’s commitment towards implementing the 3Rs of animal testing, whenever possible. While beyond the scope this paper, it notes that the incorporation of alternative approaches includes critical discussions around challenges for regulatory implementation. Building upon best practices, such as communication of NAMs through standard regulatory platforms (e.g., guidance documents) along with publications in peer-reviewed journals, presentations at conferences, and more recently through social media, will also continue to be pivotal for advancing this work.

Decades of international efforts have gone into developing legal frameworks and data requirements. While NAMs are largely in the early phases, conventional strategies such as the development of OECD guidelines, defined approaches, IATA case studies and reporting formats will continue to play a key role. Many regulators are also currently relying on testing conducted by governmental or academic research groups to develop proof of concept case studies related to the incorporation of NAMs. With established methods and acceptance criteria, broad scale testing will ultimately require industry uptake (similar to what is currently in place with traditional testing methods).

Multi-stakeholder collaboration will also continue to be important in the broader acceptance of NAMs and in enabling a better understanding of what is required for regulatory purposes. This includes initiatives led at the national level by regulatory authorities along with ensuring that the regulatory bodies continue to be engaged in key activities led by organizations such as, but not limited to the Health and Environmental Sciences Institute (HESI), PETA Science Consortium International (PSCI), and NTP Interagency Center for the Evaluation of Alternative Toxicological Methods (NICEATM). There are also several academic-led initiatives along with research and consulting firms that are immersed in developing models, which includes open source. This includes the Canadian Centre for Alternatives to Animal Methods (CCAAM) and the Canadian Centre for the Validation of Alternative Methods (CaCVAM), which aims to develop, validate, and promote non-animal, human biology-based platforms in biomedical research, education, and chemical safety testing.

There is also a need to bring all of this work together for regulatory risk assessments and decision-making. This is where frameworks, such as the Next Generation Risk Assessment as described by Krewski et al., 2014, and the recently enacted HESI committee that is responsible for the project titled Transforming the Evaluation of Agrochemicals will play a key role, in addition to other ongoing IATA and NAM-related activities at the national and global level.

## Data Availability

The original contributions presented in the study are included in the article/supplementary material, further inquiries can be directed to the corresponding author.
